# Investigating the effects of soil microstructures on bacterial growth via microfluidic channels and an agent-based model

**DOI:** 10.1038/s41598-025-23995-9

**Published:** 2025-11-17

**Authors:** Manami Ito, Ayaka Itani, Ayaka Suwa, Emi Uenaka, Kazuma Sakoda, Satoshi Sasaki, Masayuki Yamamura, Norio Takeshita, Masahiro Takinoue

**Affiliations:** 1 Department of Life Science and Technology, School of Life Science and Technology, Institute of Science Tokyo, 4259 Nagatsuta-cho, Midori-ku, Yokohama, Kanagawa 226-8501 Japan; 2 Space Environment and Energy Labs, NTT, Inc., 3-9-11 Midori-cho, Musashino-shi, Tokyo, 180-8585 Japan; 3 Microbiology Research Center for Sustainability (MiCS), Faculty of Life and Environmental Sciences, University of Tsukuba, Tsukuba, 305-8572 Japan; 4 Basic Research Labs, NTT, Inc., 3-1 Morinosato Wakamiya, Atsugi-shi, Kanagawa 243-0198 Japan; 5 Department of Computer Science, School of Computing, Institute of Science Tokyo, 4259 Nagatsuta-cho, Midori-ku, Yokohama, Kanagawa 226-8501 Japan; 6 Research Center for Autonomous Systems Materialogy (ASMat), Institute of Integrated Research, Institute of Science Tokyo, 4259 Nagatsuta-cho, Midori-ku, Yokohama, Kanagawa 226-8501 Japan; 7 Laboratory for Chemistry and Life Science, Institute of Integrated Research, Institute of Science Tokyo, 4259 Nagatsuta-cho, Midori-ku, Yokohama, Kanagawa 226-8501 Japan

**Keywords:** Ecosystem, Soil aggregates, Microfluidics, Fractal structure, Computational biophysics, Lab-on-a-chip

## Abstract

Soil is a suitable habitat for various microorganisms. These soil microorganisms establish complex social relationships and build biogeochemical cycles, such as carbon or nitrogen cycles, which synthesize plant nutrients and greenhouse gases. Clarifying microbial activities inside the soil is essential for agricultural and environmental fields. One of critical factors influencing bacterial activity in soil is the physical structure of the soil built by soil aggregates. The size distribution of soil aggregates widely ranges from µm to mm and creates pores with various sizes that act as pathways for air, water, and nutrients and affect microbial activities. Although it is known that pore size in soil is important for bacterial activity, how the pore size distribution affects bacterial activity remains unclear. The pore size distribution is considered to affect the movement of bacteria within the soil as well as that of air, water, and nutrients. Therefore, further investigation into the relationship between the size distribution of micrometer-sized pores in soil and the bacterial movement in micrometer space is required to understand bacterial activity in soil. In this study, we investigated the dependence of fractally distributed pore size distributions (from micrometers to millimeters) on bacterial activity, especially bacterial growth, using a polydimethylsiloxane culture device and numerical simulations. We fabricated a culture device with 2 μm depth with the pillars arranged fractally or periodically to represent soil particles and pore size distributions. *Escherichia coli* was cultured for 20 h in a culture device, and the results showed that final amount of *E. coli* was significantly higher in the device with the pillars arranged fractally than in the one with pillars arranged in an array. Bacterial growth was simulated in a two-dimensional space with pillars. The results also showed clear relationships among bacterial growth, movement, and pillar arrangement. Our findings provide insights into microbial activity inside complex physical structures such as soils.

**Supplementary Information:**

The online version contains supplementary material available at 10.1038/s41598-025-23995-9.

## Introduction

Soil is a habitat for various microorganisms, with an abundance of 10^7^ to 10^12^ per 1 g of soil^1^. Inside the soil, various species of microorganisms establish social relationships, such as symbiosis and competitive relationships, and build biogeochemical cycles, such as carbon or nitrogen cycles. Through these biogeochemical cycles, microorganisms synthesize plant nutrients such as nitric acid and greenhouse gases such as CO_2_, N_2_O, and CH_4_. Clarifying microbial activities inside the soil and building a numerical model of soil microorganisms are important for agricultural and environmental fields to predict plant yields or environmental impact^2–8^. These attempts have successfully achieved a certain level of predictive accuracy but are still in development, since soil is a complex system with many variables, such as the composition of chemicals or microorganisms, soil texture, and climate.

Soil has many factors, and they are complexly interrelated and impact on bacterial activity. To understand such complex system, it is essential to clarify the role of each individual factor on bacterial activity and integrate these findings. One of the critical factors that influence bacterial activity in soil is the physical structure of the soil built by soil aggregates. Soil aggregates are composed of soil particles that combine with each other, bonded by soil organic carbons, water, mycelia of fungi, plant residue, etc., inside which two to twenty times more microorganisms exist than in bulk soils^9–13^. The distribution of soil aggregates widely ranges from µm (micro-aggregates) to mm (macro-aggregates). This distributed aggregate size creates pores of various sizes that act as pathways for air, water, and nutrients^14^, and affect microbial activities^15–18^. These characteristics of aggregates, pores, and the distribution of pore size are expected to be essential factors for microbial activity in the soil.

**Fig. 1:**
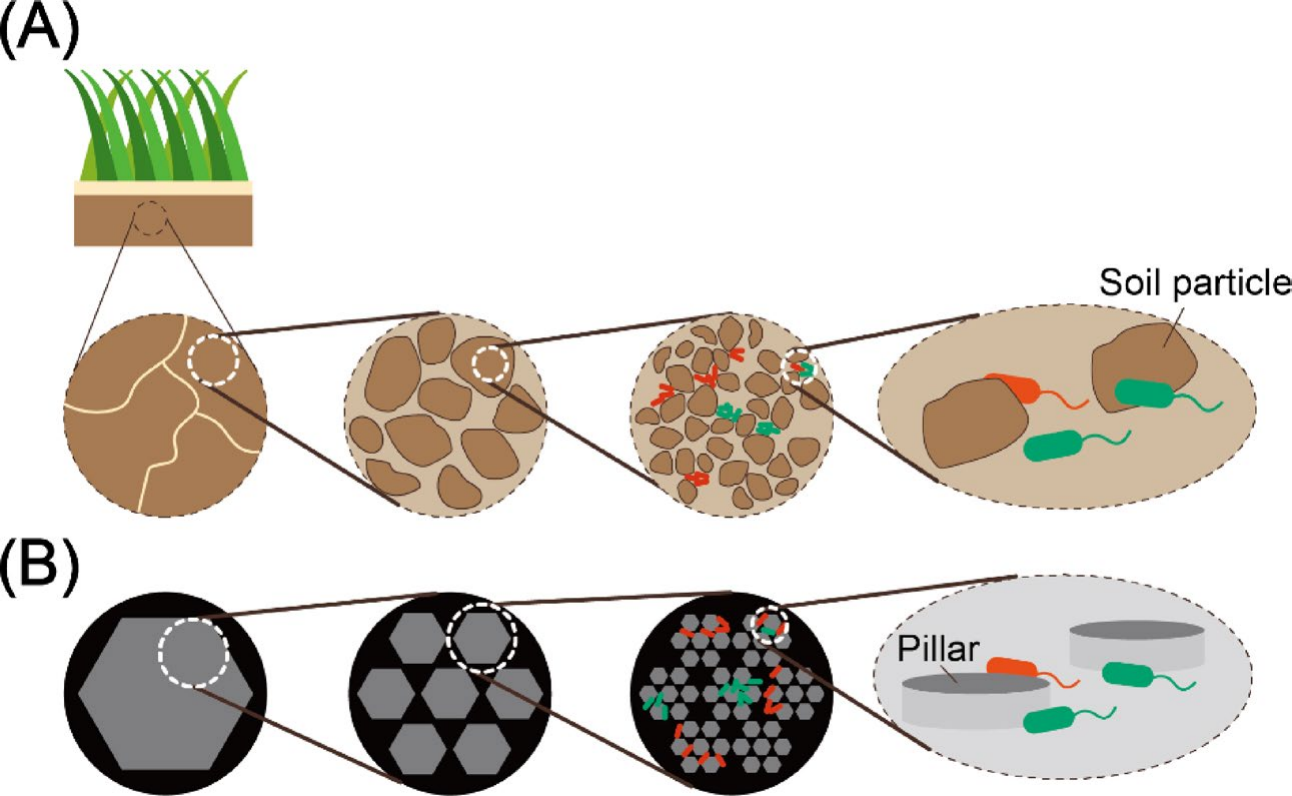
(A) Illustration of soil aggregate. The fraction of active microorganisms in aggregate is two to twenty times higher than in the bulk soil. Various sizes of aggregates form various sizes of pores. (B) We create a simulation model and microfluidic device reproducing the various sizes of pores formed by aggregates. We investigated the dependence of bacterial growth on the distribution of the size of pores with them.

The size distribution of soil aggregates reflects fractal structures^19–22^ (Fig. 1A). A fractal structure is a structure with self-similarity in different scales, i.e., structures that have similar structures at relatively large scales (e.g., mm order) and relatively small scales (e.g., µm order), which are universally found like snowflakes. Although previous studies have shown that the fractal structure of size distribution of soil aggregates is related to the soil functions^23–25^ such as water retention, the influence of fractal structure on bacterial activities remain unknown due to the difficulty of investigations. One challenge is controlling the size distribution of soil aggregates. Soil organic carbon, water, fungal mycelia, and plant residue form soil aggregates. These conditions need to be changed to control the soil aggregate size, which makes the investigation ambiguous. Furthermore, the soil is opaque to light, making it impossible to directly examine microbial activity within the soil.

To overcome these challenges, the dependence of microbial activity on the physical structure have been examined using microfluidic technologies and numerical simulations. In previous studies using microfluidic technologies, the structure within the soil or soil aggregate was simulated in space with a depth of several tens of micrometers and cultured microorganisms^26–31^. Two-dimensional numerical simulations were also used to analyze the dependence of the size and density of the physical structure on microbial activities^32–37^. These approaches provide new insights into the interactions between microorganisms and their physical structures. However, the effects of soil aggregate and pore size distributions, spanning a wide range from the size of bacteria (several micrometers) to the area size where bacteria can move (hundreds of micrometers), on bacterial activity have been poorly investigated.

In this study, we investigated the effect of the fractal size distribution of pores ranging from micrometers to millimeters on bacterial growth (Fig. 1B). We fabricated a culture chamber with a 2 μm depth with polydimethylsiloxane (PDMS). The pillars were arranged inside the culture chamber to represent soil particles and pores. We used pillar arrangements with and without a fractal structure, cultured *Escherichia coli* (*E. coli*) inside them with LB medium, and investigated their growth. We built a 2-dimensional agent-based model and simulated bacterial growth and motility within the space in which the pillars were arranged. The main objectives of this study were: (a) to investigate the effect of the fractal size distribution of pores on bacterial growth in a range of bacteria-soil interaction, ranging from several micrometers to hundreds of micrometers, using a microfluidic technique; and (b) to clarify the mechanisms by which bacterial growth is affected by the pores on that range using a numerical approach.

## Results and discussion

### Dependence of pore size distribution on bacterial growth using microfluidic devices

**Fig. 2:**
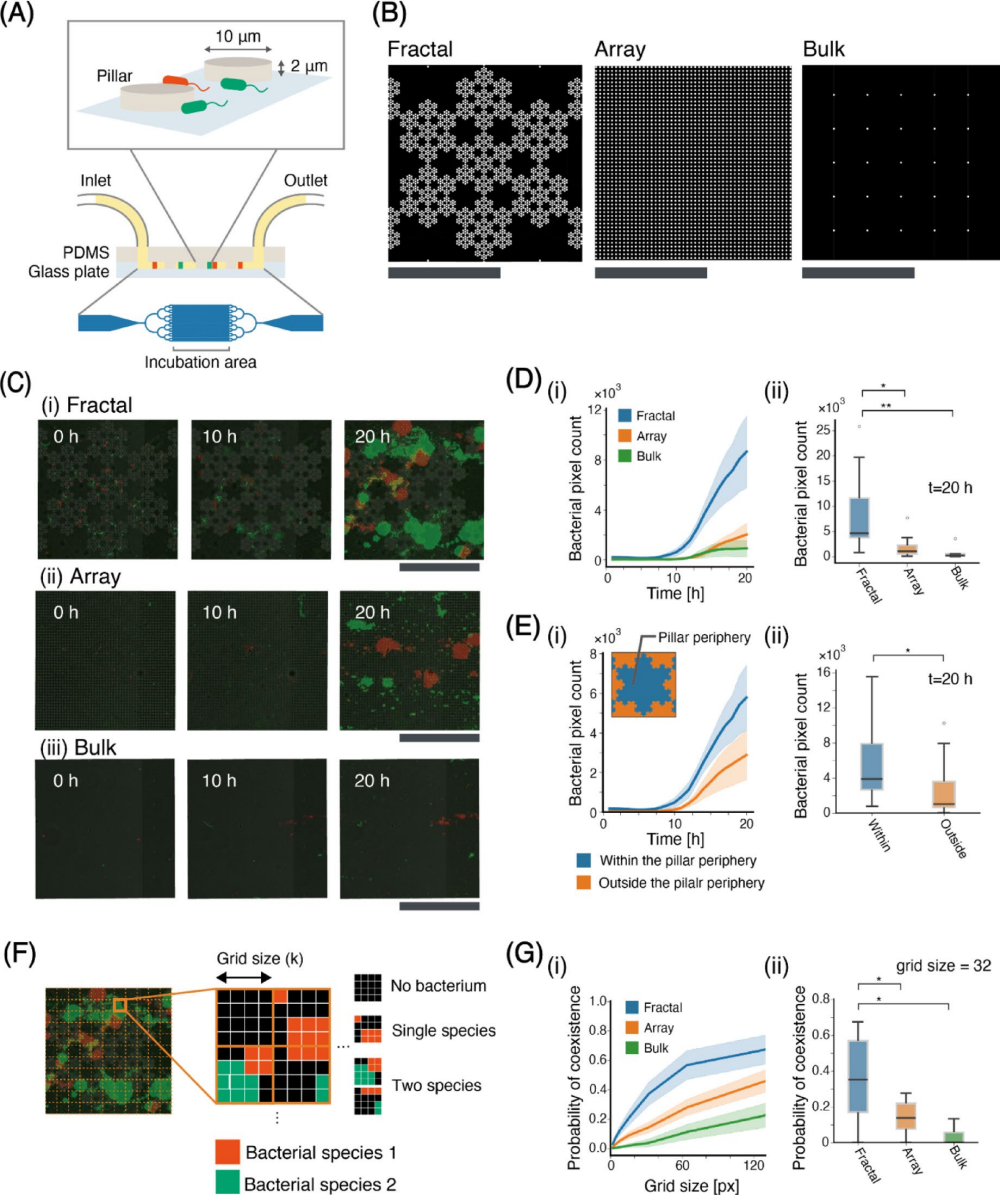
The results of verification through microfluidic devices. (A) PDMS device designs. (B)The design of the incubation chamber. The white regions represent the pillars, while the black regions indicate regions filled with LB medium, where *E. coli* can move freely. (C) Snapshots of the cultivation chamber at time 0, 10, and 20 h of (i) fractal, (ii) array, and (iii)bulk arrangements. (D) Bacterial pixel count for each arrangement. (fractal: *N* = 9, array: *N* = 8, bulk: *N* = 5) (i) Time series of bacterial pixel count for each arrangement. Error bars represent the standard error for each arrangement. (ii) box plot of bacterial pixel count at *t* = 20 for each arrangement. Box plot showing the median, interquartile range (box), and potential outliers (white-filled circle). The whiskers represent data within 1.5 times the interquartile range from the quartiles. (E) Bacterial pixel count for within and outside the pillar periphery. (*N* = 9) (i) Time series of bacterial pixel count for within and outside the pillar periphery. In the upper-left diagram in the graph, the definitions of “within pillar periphery” and “outside the pillar periphery” are illustrated. (ii) box plot of bacterial pixel count at *t* = 20 for within and outside the pillar periphery. (F) the illustration of the method for calculating the probability of both species coexisting within regions with a specific grid size. (G) the probability of both species coexisting within regions with a specific grid size. (fractal: *N* = 9, array: *N* = 8, bulk: *N* = 5) (i) the probability of both species coexisting within regions with each grid size. (ii) the boxplot of the probability of coexisting in grid size = 32, *N* = 9. Scale bars: 50 μm. Statistical significance of the Wilcoxon rank-sum test is indicated by asterisks: *P < 0.05, **P < 0.01.

We fabricated a culture device using PDMS to investigate the effects of pore size distribution on bacterial growth. The device has space 2 μm thick composed of an inlet, an outlet, and a culture chamber (Fig. 2A). Inside the culture chamber, pillars with 10 μm radius are arranged to represent soil particles. We created pore size distributions with different pillar arrangements; a region with a densely arranged pillar represented a region with small pores; conversely, a region with a sparsely arranged pillar represented a region with large pores. Three types of pillar arrangements were fabricated: fractal, array, and bulk (Fig. 2B). Fractal arrangement refers to pillars fractally arranged, with the shortest distance between pillars of 2 μm, representing soil aggregates. An array arrangement is an arrangement in which approximately the same number of pillars as fractal arrangement are uniformly arranged, representing a condition without soil aggregates. The bulk arrangement has the minimum number of pillars, serving as a control experiment; the pillars were placed only to prevent the deflection of PDMS.

We cultured *E. coli* in the culture device. *E. coli* was chosen because it is found in soil, and its motility and readily simple growth characteristics are suitable for testing the feasibility of our device (Note that other microorganisms could be used as test species; *E. coli* was chosen as a first step). We used two strains of *E. coli* with green and red fluorescent proteins (mClover and mScarlet, respectively). To focus on the relationship between the physical structure and bacterial growth, we chose strains without inhibition or symbiosis, i.e., two strains that had no interaction except for nutrient and space competition. Each *E. coli* strain was cultured separately overnight, diluted 100 times with LB medium, and mixed. The mixed culture was introduced into the culture chamber through a tube connected to an inlet. The device was placed under a microscope with a hot bath and incubated for 20 h. Tubes filled with LB medium were connected to an inlet and outlet to prevent drying.

The bacterial growth in the device (fractal, array, and bulk) was monitored by time-lapse imaging every hour for total 20 h (Fig. 2C, Supplementary Movies S1-S3). The light gray area is the pillar, the red or green area is the region where *E. coli* with mClover or mScarlet exists, and the dark gray area is the area filled with LB medium. The snapshots show that in the fractal and array arrangements, both mClover and mScarlet strains of *E. coli* successfully grew, and the amount of *E. coli* was higher in the fractal than in the array at 20 h. The snapshots also show the spatially heterogeneous existence in the fractal arrangement. *E. coli* tended to exist near the pillar region. In a fractal arrangement, the strains, mClover and mScarlet, were spatially well mixed compared to array arrangement.

The bacterial growth was compared by the bacterial pixel counts for each pillar arrangement (Fig. 2D). We counted the red or green pixels, which refer to bacterial pixels, as the amount of *E. coli*^38^. Figure 2D(i) shows the time series of the bacterial pixel counts for each pillar arrangement. In the fractal arrangement, the bacterial pixel count started to increase at approximately 5 h. and finally reaches 12,000 px after 20 h. On the other hand, in the array and bulk arrangements, the bacterial pixel count started to increase at approximately 10 h but reached less than 2,000 px at 20 h. Figure 2D(ii) shows a boxplot of the bacterial pixel counts at 20 h for each arrangement. Although a distribution of bacterial pixel counts for the fractal arrangement is wide, there was statistically significant difference in bacterial pixel count between the array/bulk and fractal arrangements (Wilcoxon rank-sum test, *P* < 0.05). There was no statistically significant difference in the bacterial pixel count between the array and bulk arrangements (Wilcoxon rank-sum test, *P* = 0.28).

Figure 2E shows the bacterial pixel counts in the fractal arrangement within the pillar periphery and outside the pillar periphery. A definition of the pillar periphery is shown by the inset of Fig. 2E(i). Figure 2E(i) shows the time series of bacterial pixel counts in each region. Within the pillar periphery, the bacterial pixel count started to increase at approximately 7.5 h and finally reached 6, 000 pixels at 20 h. Conversely, outside the pillar periphery, the bacterial pixel count started to increase around 10 h but reached approximately 3,000 px at 20 h. Figure 2E(ii) shows a boxplot of the bacterial pixel counts at 20 h for each region. Owing to the wide distribution of fractals, there was no statistically significant difference in the bacterial pixel count between regions (Wilcoxon rank-sum test, *P* = 0.07). The median bacterial pixel count for the region within the pillar periphery was notably larger than that in the region outside the pillar periphery.

We investigated the degree of mixing between the two strains. To examine the degree of mixing, we divided the culture region into *k*×*k* grid sizes (grid size: *k* = 2, 4,16, 32, 64, and 128) and calculated the proportion of grids containing both strains for at least one strains (Fig. 2F and G). In other words, we assumed that a high degree of mixing could translate to a high probability of coexistence within a small grid.

Figure 2G(i) shows the probability of the coexistence of each grid size and pillar arrangement. The results showed that the probability of coexistence was higher in the fractal arrangement for all grid sizes, followed by array and bulk arrangements. Figure 2G(ii) presents a boxplot of the probability of coexistence when the grid size is 32 for each arrangement. Although the distribution of the probability of coexistence for fractal arrangements was wide, there was a statistically significant difference between fractals and the other arrangements (Wilcoxon rank-sum test, *P* < 0.05).

These results show that the bacterial growth is affected by the pillar arrangement. A previous study using PDMS reported that the bacteria tend to increase within a region where obstacles aggregated, which was explained by the increased fluid shear within such regions^26^. Our results show a notable increase in bacterial pixel count within a pillar periphery in a fractal arrangement. However, in our experimental settings, the tubes filled with LB medium inserted into the inlet and outlet, so continuous flow in a culture chamber was not assumed. Thus, other mechanisms may contribute to increase in bacterial pixel in a fractal arrangement.

### Agent-based simulation model

**Fig. 3:**
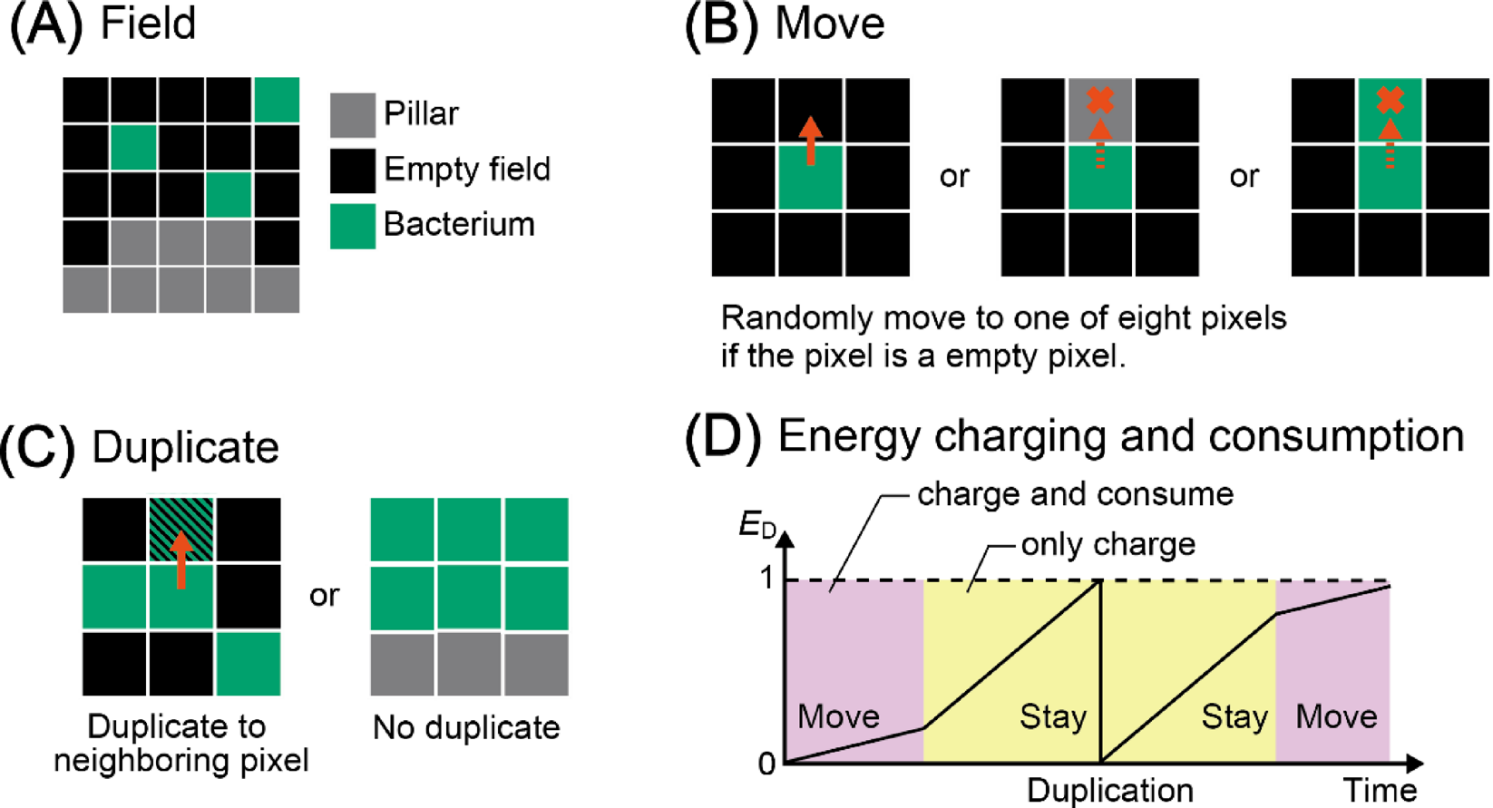
The simulation model of this study. (A) Field composition. A bacterium is represented by an agent with one pixel and moves on a 2D field with 256 × 256 pixels. The field is composed of empty field pixels and pillar pixels. (B) The bacterium randomly selects one of eight neighboring pixels and moves to the selected pixel if the selected pixel is an empty pixel. (C) A bacterium duplicates if an empty pixel exists in the neighboring 8 pixels. (D) The timing of the duplication is managed by a value of the energy required for duplication, *E*_D_ (0 ≦ *E*_D_≦1). *E*_D_ is constantly increasing up to 1; the bacterial movement reduces *E*_D_. A bacterium duplicates when *E*_D_ reaches 1, and *E*_D_ is reset to 0 just after the duplication.

Experimental investigations using microfluidic devices suggest that pillar arrangement influences bacterial growth. To examine the dependence of pillar arrangement on bacterial growth, we constructed a two-dimensional (2D) agent-based model (Fig. 3). As non-interacting bacteria were used in the experiment, we conducted simulations using a single type of bacterium. This simplified model allowed us to focus on the relationship between bacterial movement and pore size. A bacterium is represented by an agent with one pixel, called a bacterial pixel, and moves in a 2D field of 256 × 256 pixels. The field comprised empty field pixels representing pores inside the soil and pillar pixels representing soil particles (Fig. 3A). We assumed that a bacterium moves randomly; randomly selected one of eight neighboring pixels to move on (Fig. 3B). The bacterium moves to the selected pixel if the selected pixel is not a pillar pixel or another bacterial pixel. This condition, in which a bacterium does not move to other bacterial pixels, leads to spatial competition among bacteria.

A bacterium duplicates if an empty pixel exists among eight neighboring pixels (Fig. 3C). Through bacterial duplication, a bacterium occupies a neighboring pixel; the newly occupied pixel is randomly selected from the neighboring empty pixels. The timing of the duplication is managed by a value of the energy required for duplication, *E*_D_ (0 ≤ *E*_D_≤1). *E*_D_ continuously increased to 1 at the growth rate. We introduced energy consumption as the bacteria move, as previous studies have indicated that bacterial movement consumes energy and reduces growth^39,40^. Bacterial movement reduced *E*_D_ by an energy consumption at an energy consumption rate of (Fig. 3D, Supplementary Fig. S4A). A bacterium duplicates when the *E*_D_ reaches 1 and is reset to 0 immediately after duplication. This assumption indicates that actively moving bacteria duplicate less frequently than those that do not. Stochastic bacterial death was introduced. Sufficient nutrients are assumed to allow the bacteria to continue their movement and duplication.

As in the microfluidic experiments, we first prepared the three-pillar arrangements (fractal, array, and bulk). A pillar was defined as a group of circular pillar pixels with a radius of 6 px. The fractal arrangement is a fractal pillar arrangement with fractally arranged pillars having the shortest distance between pillars of 3 px, and the array arrangement is a periodic array of pillars with the same number of pillars as the fractal arrangement. The bulk arrangement was prepared as a control, in which the pillars were not placed.

### Dependence of pore-size distribution on bacterial growth using simulations

**Fig. 4:**
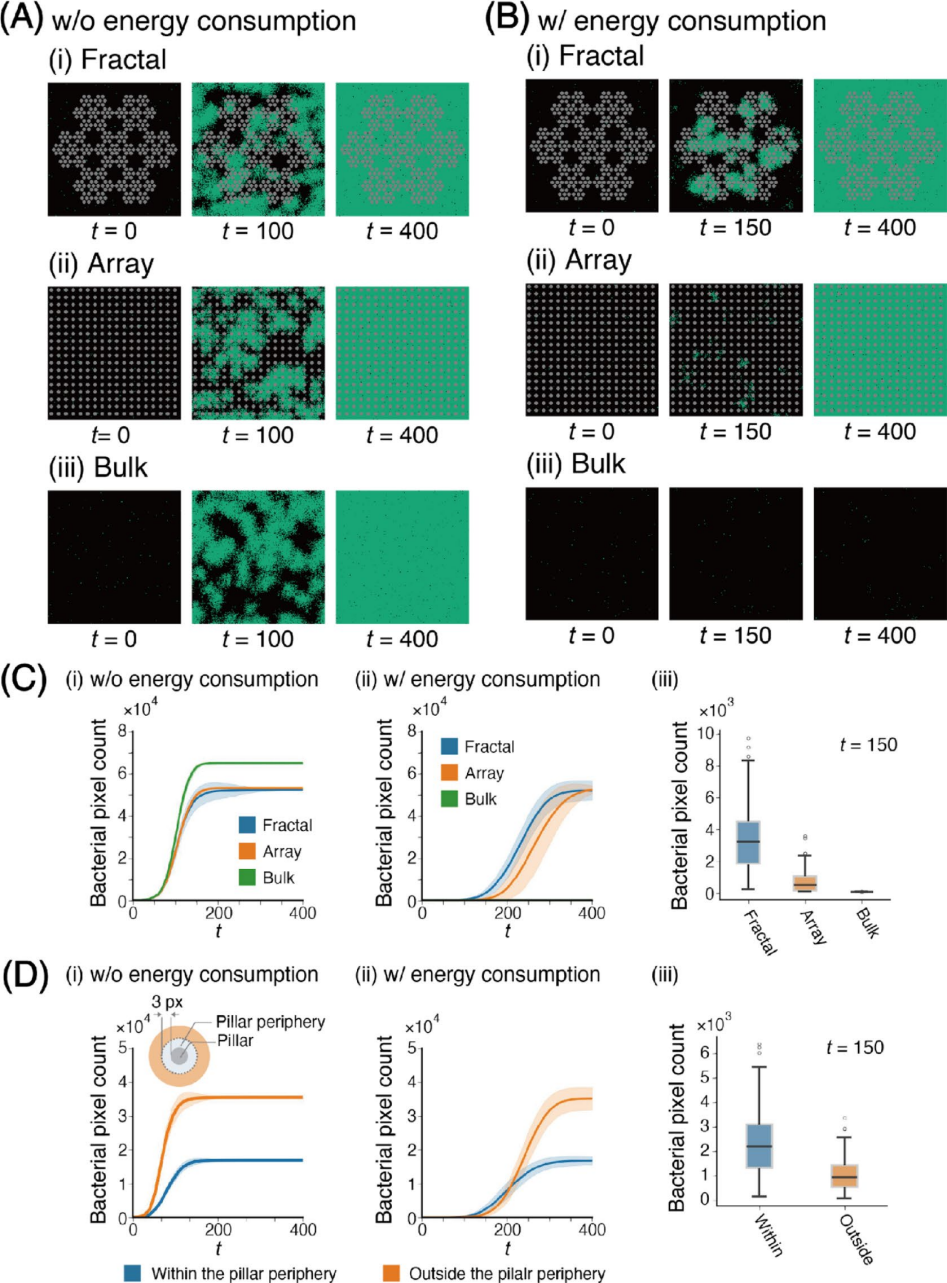
Simulation results for fractal, array, and bulk arrangements. (A) Snapshots at simulation time steps *t* = 0, *t* = 100, and = 400 for simulations conducted without energy consumption: (i) fractal, (ii) array, and (iii) bulk arrangements. (B) Snapshots at simulation time steps *t* = 0, *t* = 100, and *t* = 400 for simulations conducted with energy consumption: (i) fractal, (ii) array, and (iii)bulk arrangements. (C-D) Analytical results from 100 simulation runs: (C) Time series of bacterial pixel counts for fractal, array, and bulk arrangements under (i) no energy consumption and (ii) energy consumption conditions. (iii) Boxplot of bacterial pixel counts at *t* = 150 for each arrangement with energy consumption. (D) Time series of bacterial pixel counts within and outside the pillar periphery for fractal arrangement under (i) no energy consumption and (ii) energy consumption conditions. (iii) Boxplot of bacterial pixel counts at *t* = 150 for each region with energy consumption. The definition of the pillar periphery is illustrated in (D)(i). Statistical significance of the Wilcoxon rank-sum test is indicated by asterisks: *P < 0.05, **P < 0.01.

Figure 4A shows snapshots of the bacterial pixel growth simulation results for three different arrangements (fractal, array, and bulk) under conditions in which bacteria grow without energy consumption (Supplementary Movies S4-S6). A snapshot of the fractal arrangement at *t* = 100 shows that the bacterial pixel expansion begins at multiple locations, independent of the presence of pillars (Fig. 4A(i)). Similarly, snapshots of the array and bulk arrangements also showed bacterial growth initiating at multiple locations, as observed in the fractal arrangement (Figs. 4A(ii) and (iii)). Moreover, there was no significant difference in bacterial pixel counts among the different pillar arrangements.

Figure 4B shows snapshots of the bacterial pixel growth simulation results for three different arrangements (fractal, array, and bulk) under conditions in which bacteria grow with energy consumption (Supplementary Movies S7-S9). In contrast to Fig. 4A(i), the snapshot of the fractal arrangement at *t* = 150 shows that bacterial pixel expansion occurred around the periphery of the pillars. The snapshots of the array arrangement show a smaller bacterial pixel count at *t* = 150 compared with the fractal arrangement. However, the bacterial pixel count increased over time. In the bulk arrangement (Fig. 4B(iii)), the bacterial pixel count did not increase with time.

Because the initial positions of the bacteria were random, the results of the growth curves varied for each simulation. Therefore, we ran 100 simulations to analyze the results statistically. Figure 4C(i) shows the growth curve of bacteria under conditions in which the bacteria grew without energy consumption in each arrangement. The growth curve of all arrangements saturates at *t* = 150. Saturation means that the bacterial pixels fill all fields. The bacterial pixel count at the saturation point is higher in the bulk arrangement. However, that of fractal and array arrangements are approximately the same because the bulk arrangement does not have a pillar where bacteria cannot expand, and the fractal and array have approximately the same number of pillars. Figure 4C(ii) shows the growth curve of the bacteria under conditions in which the bacteria grow with energy consumption in each arrangement.

In contrast to the growth without energy consumption, the bacterial pixel count in the bulk arrangement did not increase. Meanwhile, the bacterial pixel count increased from approximately *t* = 200 and saturated at 300 < *t* < 400 in the fractal and array arrangements. The increase in bacterial pixel count in the fractal arrangement was faster than that in the array arrangement. Figure 4C(iii) presents a boxplot of the bacterial pixel count at *t* = 150 for each arrangement. The distribution of bacterial pixel counts in the fractal arrangement was broad, with a significant difference compared with the array (Wilcoxon rank-sum test, *P* < 0.05). Similarly, the difference in bacterial pixel counts between the array and bulk arrangement was significant.

Figure 4D(i) shows the difference in the bacterial pixel count between the inside and outside of the pillar periphery under conditions where bacteria grow without energy consumption in the fractal arrangement. The periphery of the pillar is shown in the inset of Fig. 4D (i). The results indicate that bacterial growth outside the pillar periphery is slower but eventually saturates at *t* = 150, reaching approximately twice the bacterial pixel count compared to the inside. This difference at saturation reflects the variation in the size of the two regions. Figure 4D(ii) shows the same comparison under conditions where bacteria grow with energy consumption in the Fractal pattern. The results show that, while the bacterial pixel count outside the pillar periphery again saturates at = 300 with about twice the bacterial pixel count, the bacterial pixel count is smaller at *t* < 200, indicating a slower growth rate than the inside of the periphery. Figure 4D(iii) shows the boxplot of bacterial pixel count at *t* = 150 under conditions where bacteria grow with energy consumption within and outside the pillar periphery. The results show that the bacterial pixel count within the pillar periphery has a more extensive distribution and is significantly higher than that outside the periphery.

These results show that bacterial pixel count increases faster in the fractal arrangement than in the array arrangement only under conditions in which bacteria grow with energy consumption. This demonstrates that phenomena like those observed in the experiments can also be observed in the simulations under conditions in which bacteria grow with energy consumption. These findings suggest that energy consumption is a key factor in the relationship between pore size distribution and bacterial growth.

Since our simulation settings do not include medium flow, the difference in bacterial growth among the pillar arrangements is driven solely by microbial motility and energy consumption. The results also show that bacterial pixel accumulation begins near the pillars, which allows us to hypothesize that the pillar arrangement influences microbial motility and energy consumption.

### Dependence of bacterial growth on the difference of the pillar-dense region

**Fig. 5:**
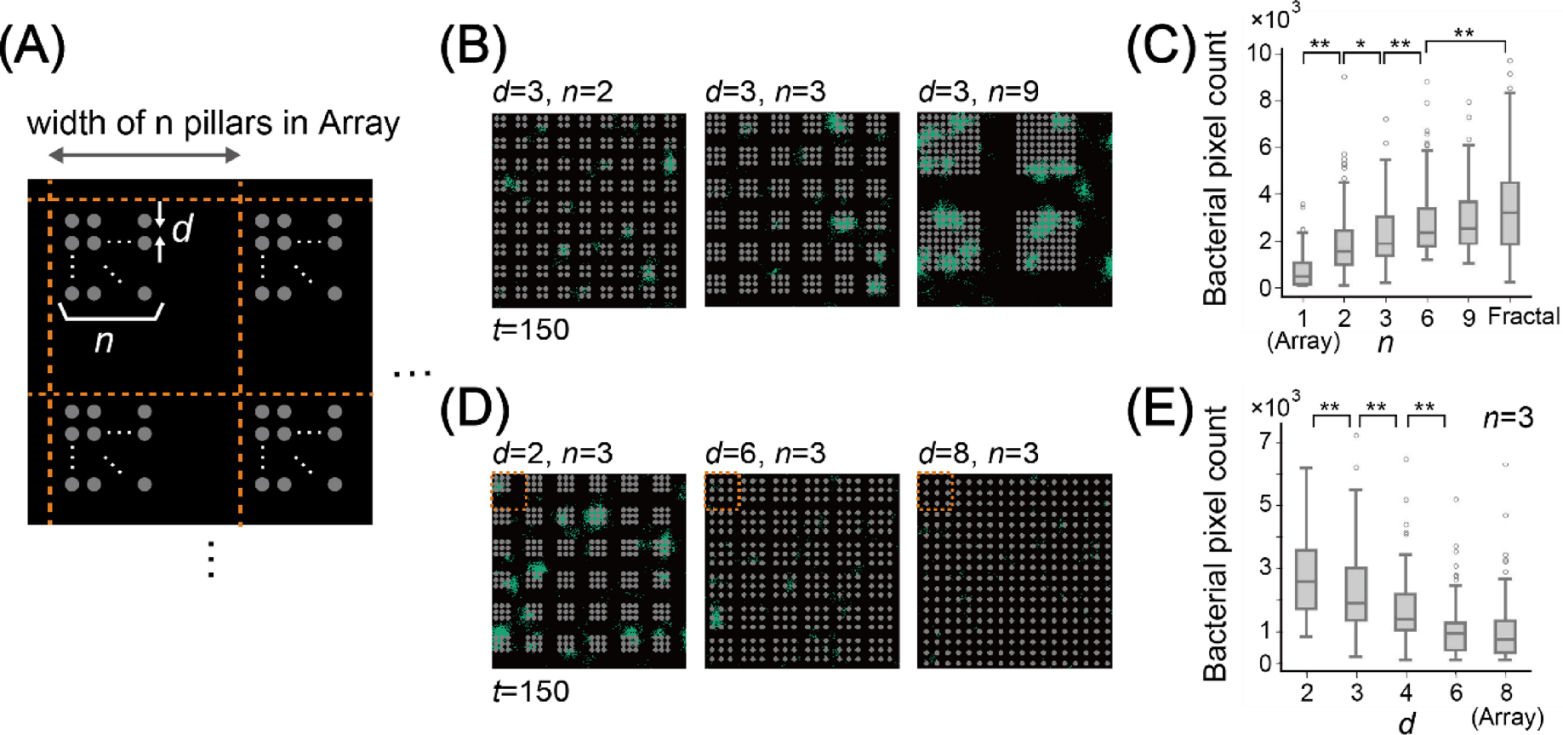
Simulation results of bacterial growth in arrangements with varying the densely packed pillar region sizes and densities. (A) Definition of the distance between pillars (*d*) and the size of the densely packed pillar region (*n*). (B) Examples of arrangements with varying *n* (*d* = 3) at *t* = 150. (C) Boxplot of bacterial pixel counts for each arrangement with varying *n* (*d* = 3) and fractal arrangement at *t* = 150. (D) Examples of arrangements with varying *d* (*n* = 3) at *t* = 150. (E) Boxplot of bacterial pixel counts for each arrangement with varying *d* (*n* = 3) at *t* = 150. Results are based on 100 simulation runs. Statistical significance of the Wilcoxon rank-sum test is indicated by asterisks: *P < 0.05, **P < 0.01.

The results of both microfluidic experiments and numerical simulations show a faster increase in the bacterial pixel count in the fractal arrangement than in array arrangement. The main difference between the array and fractal arrangements is the presence of a dense pillar region. We further investigated the effect of the pillar-dense region on the increase in the bacterial pixel count with block-like arrangements. Block-like arrangements have periodically arranged pillar blocks, within which *n*×*n* (*n* = 1, 2, 3, 6, and 9) pillars are arranged squarely (Figs. 5A and Supplementary Movies S10-S13). Each block-like arrangement has the same number of pillars as the array arrangement, and the block-like arrangement with *n* = 1 has the same arrangement as the array arrangement. Figure 5B shows snapshots of bacterial growth at *t* = 150 for various *n*. The snapshots show a tendency for the existence of more bacterial pixels in the larger n and pillar-dense regions. Figure 5C presents a boxplot of the bacterial pixel count at *t* = 150 in a block-like arrangement for each *n*. When *n* = 1, the median bacterial pixel count was approximately 500; as *n* increased, the median bacterial pixel count also increased. The median bacterial pixel count reached approximately 2,500 when *n* = 9. However, the median bacterial pixel count of the fractal arrangement was approximately 3,000, which was still larger than *n* = 9. These results show the dependency of the pillar-dense area size on the increase in bacterial pixel count.

We further investigated the dependence of the distance between pillars (*d*) within the block, i.e., the density of pillars within the block, on the bacterial pixel count (Fig. 5A). Note that the arrangement with *d* = 8 was the same as that of the array arrangement. Figure 5D shows an example of the snapshot at *t* = 150 with various d values and *n* = 3 (Supplementary Movies S14-S17). The snapshots show the tendency of the existence of more bacterial pixels in a smaller *d*. Figure 5E shows a boxplot of the bacterial pixel count at *t* = 150 in block-like arrangements for each *d*. When *d* = 2, the median bacterial pixel count was approximately 2,500, and as *d* increased, the median bacterial pixel count decreased. The median bacterial pixel count with *d* = 2 is lower than that of the fractal arrangement. A similar result was observed for another *n* (*n* = 2, 6, 9; see Supplementary Fig. S1).

These results indicate that the size and density of pillar-dense region affect the bacterial growth. Previous studies have shown that the region with small pores, like pillar-dense region, promote compartmentalization and coexistence of bacteria, and the density of small pores is a key factor for coexistence. Our results further suggest that the density of small pores is crucial for bacterial growth itself, and that the size of these regions may also be a key factor.

### Differences in bacterial movement depending on pillar arrangements

We further examined the difference in contact between the pillar and bacteria among the pillar arrangements to investigate the difference in the impact of the pillar on bacteria among the pillar arrangements. We randomly placed a bacterium without death and duplication in each pillar arrangement, simulated the replacement for 0 ≤ *t* ≤ 500, and analyzed the proportion of the time spent outside the pillar periphery (*p*_outside_). We defined the pillar periphery as pixels within three pixels apart from the pillars within 3 pixels, as illustrated in Fig. 4D.

**Fig. 6:**
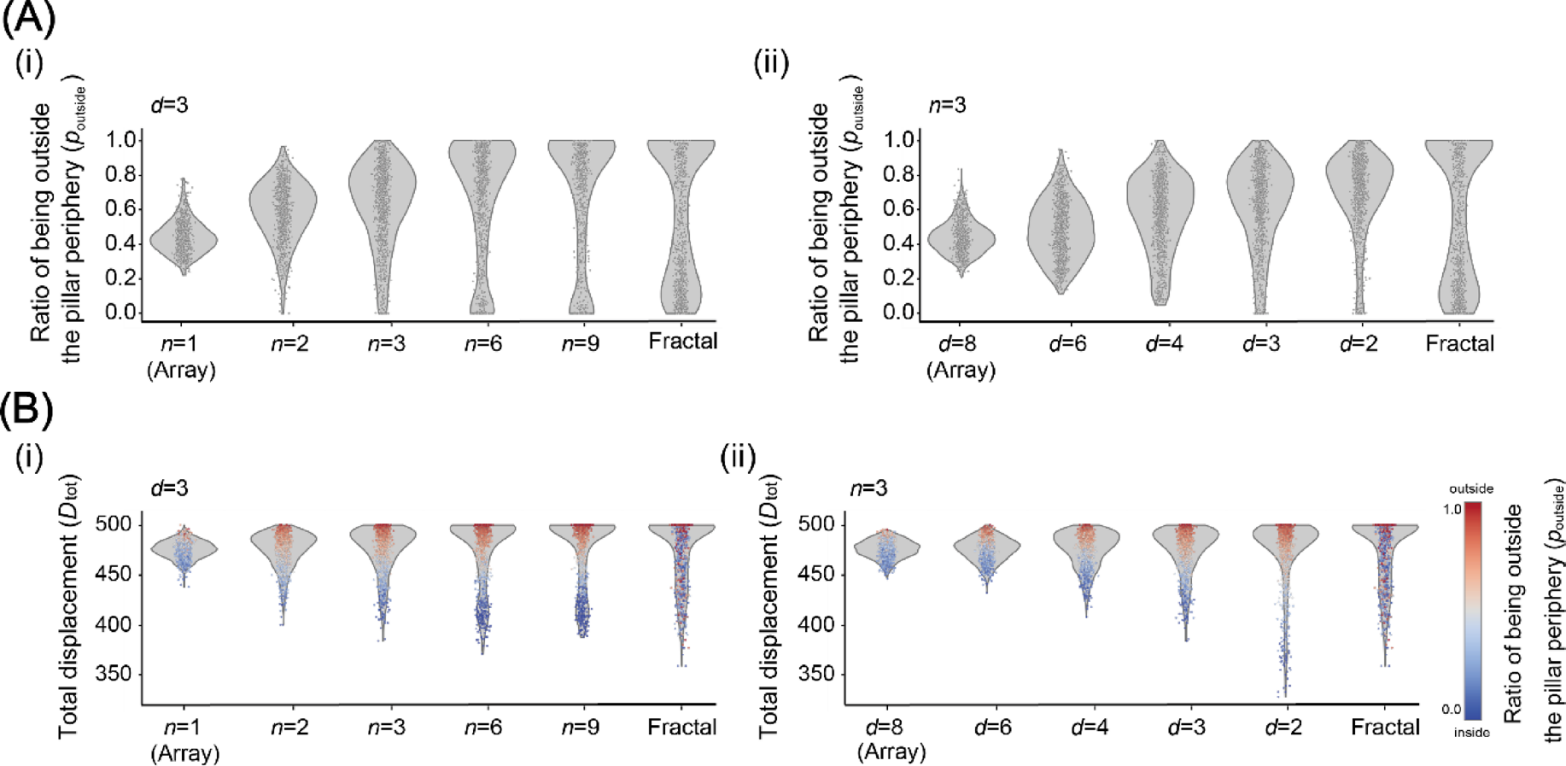
(A) The violin plot of the distribution of the ratio of the time that each bacterium is outside the pillar periphery to the total simulation time (*p*_outside_) for 0 < *t* < 500 of each arrangement (i) *n* = 1, 2, 3, 6, and 9 and fractal arrangement, *d* = 3 and (ii) *n* = 1, 2, 3, 6, and 9 and fractal arrangement, *d* = 3). (B) The violin plot of distribution of the total displacements of bacteria (*D*_tot_) of each arrangement (i) (*n* = 1, 2, 3, 6, and 9 and fractal arrangement, *n* = 3 and (ii) *d* = 1, 2, 3, 6, and 9 and fractal arrangement, *n* = 3. The colors of the dots represent *p*_outside_. Results are based on 1000 simulation runs.

Figure 6A shows the violin plot of *p*_outside_ for 0 ≤ *t* ≤ 500 in block-like arrangements for each *n* and fractal arrangement (*d* = 3, see Fig. 5B for details about pillar arrangements). Each dot represents *p*_outside_ of each bacterium for the 1000 simulation runs. When *n* = 1, *p*_outside_ distributes from 0.2 to 0.8 and has a peak around 0.4. As *n* increases, the distribution of *p*_outside_ becomes wider, and the peak becomes higher: 0.7 for *n* = 2, 0.8 for *n* = 3, and exceeds 0.9 for *n* = 6 and 9. For *n* = 6 and 9 and the fractal arrangement, the second peak was observed from 0 to 0.2. The second peak of the fractal arrangement was larger and wider than the others. Figure 6B shows the violin plot of *p*_outside_ for 0 ≤ *t* ≤ 500 in block-like arrangements for each *d* and fractal arrangement (*n* = 3; see Fig. 5D for details about pillar arrangements). When *d* = 8, *p*_outside_ distributes from 0.2 to 0.8 and has a peak at approximately 0.4, similar to *n* = 1 in Fig. 6A. As *d* decreases, the distribution of *p*_outside_ becomes wider, and the peak becomes higher; 0.7 for *d* = 4 and exceeds 0.8 for *n* = 3, 2 and exceeds 0.9 for *n* = 6 and 9. In contrast to the arrangement with larger *n*, the second peak was not observed for any *d*.

These results indicate that the bacterial count with smaller *p*_*outside*_, i.e., relatively less contact with pillars, increased in arrangements with larger *n*, smaller *d*, and fractal arrangement. This condition of a larger *n* and smaller *d* is consistent with the condition of the arrangement with a faster increase in the bacterial pixel count. We further examined the total displacement (*D*_tot_) of each bacterial pixel for 500 simulation time steps to investigate the relationship between the bacterial pixel increase and *p*_outside_ As *D*_tot_ reflects the mobility of bacteria and is under conditions of energy consumption and movement, *D*_tot_ affects the efficiency of bacterial duplication (Fig. 3D). *D*_*t*ot_ is the total movement count and not the distance between the initial and final positions.

Figure 6B shows the violin plot of *D*_tot_ for 0 ≤ *t* ≤ 500 in block-like arrangements for each *n* and fractal arrangement (*d* = 3; see Fig. 5B for details about pillar arrangements). Each dot represents the *D*_tot_ of each bacterium for 1000 simulation runs, and the color of the dots represents *p*_outside_. The bacterial pixels were moved by 0 or 1 pixel for each simulation time step. The maximum value of *D*_tot_ was 500. When *n* = 1, *D*_tot_ was distributed from 470 to 490; the dots around *D*_tot_ = 490 tended to have a larger *p*_outside_ (red), and the dots around *D*_tot_ = 700 tended to have a smaller *p*_outside_ (blue). As *n* increases, the distribution of *D*_tot_ widens and the peak of *D*_tot_ increases, similar to the violin plot of *p*_outside_. The distribution of *p*_outside_ becomes bipolar as the distribution of *D*_tot_ widens; dots 470 < *D*_tot_ tend to have a large *p*_outside_ (red), and dots *D*_tot_ < 430 tend to have a small *p*_outside_ (blue). Figure 6D shows the violin plot of *D*_tot_ for 0 ≤ *t* ≤ 500 in block-like arrangements for each *d* and fractal arrangement (*n* = 3; see Fig. 5D for details about pillar arrangements). When *d* = 8, *D*_tot_ is distributed from 470 to 490, and as *n* increases, the distribution of *D*_tot_ widens and the peak of *D*_tot_ increases, similar to the violin plot of *p*_outside_. The distribution of *p*_outside_ becomes bipolar as the distribution of *D*_tot_ becomes wider; dots 470 < *D*_tot_ tend to have a large *p*_outside_ (red) and dots *D*_tot_ < 430 tend to have a small *p*_outside_ (blue), similar to the violin plot of *D*_tot_ with various *n*. While the distribution of *p*_outside_ for block-like arrangements shows a clear bipolar, *p*_outside_ for the fractal arrangement distribute widely. We calculated the correlation between *p*_outside_ and *D*_tot_ for each arrangement, which showed a strong correlation for block-like arrangements, but no correlation was found between the two in the fractal arrangement (Supplementary Fig. S2).

These results indicate that the size. and density of pillar-dense region affect the bacterial motility, resulting in bacterial growth. In the block-like pillar arrangements, bacteria polarize into two groups; bacteria trapped within the block-like region and restricted their movement, and bacteria unable to enter the block-like region and feely moved. On the other hand, in the fractal pillar arrangements, such polarization cannot be observed; bacteria move regardless of the presence of the pillar, that may cause by the pillar-dense area and the other area are fractally mixed.

## Conclusion

In this study, we investigated the dependence of fractally distributed pore size distributions from micrometers to millimeters on bacterial growth using a PDMS microfluidic device and numerical simulations. We fabricated a PDMS device with three types of pillar arrangements: a fractally distributed pillar arrangement (fractal), a periodically distributed pillar arrangement (array), and an arrangement with no pillars (bulk). *E. coli* was cultured for 20 h in a culture device, and the results revealed that the amount and mixture degree of *E. coli* were higher in the fractal arrangement than array and bulk arrangements. The results also show that in fractal arrangements, the amount of *E. coli* increases around the pillar region, which exists densely. Focusing on the difference in the existence of pillar-dense areas between the fractal and array arrangements, we further investigated the block-like arrangement with various block sizes and distances between pillars. Simulation results show that block-like arrangements with shorter distances between pillars and larger blocks show a faster bacterial pixel increase. Nevertheless, in fractal arrangements, it is faster. We also examined bacterial movement in each arrangement. The results showed a clear tendency of bacterial pixels to restrict their movement by pillars in block-like arrangements, with shorter distances between pillars and larger blocks. The results also showed that a more complex movement in the fractal arrangement is the key factor for the faster increase in bacterial pixels. The effects of distributed pore sizes were not focused on in the previous study^32^, which investigated differences in bacterial activity within pores of varying sizes. In this study, we investigated how the distributed pore sizes affect bacterial growth, and our simulation results suggest that this distribution may be an important factor influencing bacterial behavior.

A previous study using PDMS reported that the bacteria tend to increase within a region where obstacles aggregated, which was explained by the increased fluid shear within such regions^26^. Similarly, our results show a notable increase in bacterial pixel count within a pillar periphery in a fractal arrangement. However, our experimental and simulation settings do not include medium flow, indicating that the differences in bacterial growth among the pillar arrangements are driven solely by microbial motility and energy consumption. Our result yield additional insight to the dependence of the size distribution of pores on bacterial growth.

Our microfluidic experimental and numerical simulation settings were highly simplified, focusing on the interactions between the physical structure and bacterial movement, which provided clear insights into the relationship between them. However, realistic soil is more complex, in which diverse bacteria have various social interactions (i.e., competition, prey-predator, symbiosis, and inhibition), morphologies (e.g., bacilli, cocci, and filamentous fungi), and movements. Previous reports have emphasized the importance of these features in a bacterial activity. Introducing these features into our experimental settings will provide new insights into soil bacterial activities. Additionally, we used *E. coli*, which rarely exists in soil, in the microfluidic experiments. Introducing soil microorganisms through isolation or soil extraction yields results that are closer to the realistic environment of the soil.

In the fields of agriculture and environment, researchers have studied methods for inoculating useful microorganisms into soil and improving their functions^41–44^. Porous materials that effectively carry and introduce microorganisms into the soil have been studied. Clarifying the relationship between physical structure and bacterial activity would offer important insights into these studies, as well as the modeling and predictions of biochemical cycles.

## Methods

### Device design

The device was designed in Illustrator 2021 (Adobe, San Jose, CA, USA). The design consisted of three parts: an inlet/outlet where microorganisms were inoculated, and a cultivation chamber where bacteria were cultured. The inlet/outlet had a bifurcated structure to uniformly inoculate the bacteria and nutrients. The thickness of the cultivation chamber is 2 μm. The pillar arrangement of the cultivation chamber had three variations: fractally arranged pillars (fractal) and aligned pillars (array and bulk). All arrangements had the same area. It was ensured that the fractal arrangement and arrays contained approximately the same number of pillars. However, owing to constraints, such as the aspect ratio of the area, achieving an exact match was not possible (fractal: 7,555; array: 6,656). The fractal dimension (Hausdorff dimension) calculated using the box counting method of the fractal arrangement is approximately 1.81, which is close to the fractal dimension observed in soil cross-sections obtained by scanning electron microscope in a previous study (1.58 to 1.91)^45^.

### Device fabrication

The microfluidic system was molded using PDMS. The mold was made on a heat-dried (200℃ for 10 min) 2-inch silicon wafer (GA2002, MCO, Kanagawa, Japan). SU-8 3005 (MicroChem, Newton, MA, USA) was spin-coated on the wafer at 3000 rpm to get a 2 μm thick layer and soft-baked (95℃ for 15 min after 65℃ for 5 min). Subsequently, the pattern was projected onto an SU-8 layer on a wafer using a maskless photolithography system (D-Light DL-1000, Nano System Solutions, Okinawa, Japan). After UV exposure, the wafer was post-baked at 95℃ for 15 min and developed with the SU-8 developer. The wafer was put on a dish, and PDMS (SYLGARD™ 184 Silicone Elastomer, Dow, Midland, MI, USA) was poured into it. The PDMS was deaerated and baked at 65℃ for 1.5 h on a hotplate. After the PDMS was cut from the mold, inlets/outlets were made using a biopsy punch, and the PDMS was bonded to a glass plate using a plasma treatment.

### Culture

To help microorganisms inoculate uniformly, the cultivation chamber was filled with LB medium prepared with the following components: Bacto™ Yeast Extract (Gibco, Thermo Fisher Scientific, Waltham, MA, USA), Bacto™ Tryptone (Gibco, Thermo Fisher Scientific, Waltham, MA, USA), and NaCl (Wako, Osaka, Japan). PE tubes (INTRAMEDIC™ Polyethylene Tubing, Thermo Fisher Scientific, Waltham, MA, USA) were inserted into the inlet/outlet, and LB medium was injected using a syringe.

Two strains of *E. coli* DH5α with pMEX9-Plac-mScarlet-I-Pa or.

pMEX9-Plac-mClover3-Pa were pre-cultured in an LB medium with 100 µg/mL Gentamicin (Fujifilm, Kyoto, Japan) overnight. These cultures were diluted 100 times with LB medium and mixed. Cultures containing the two strains were inoculated into a cultivation chamber through a PE tube using a syringe. To prevent drying, we inserted PE tubes filled with the LB medium until the experiment was complete. Microfluidic system was put on a hotplate with a hot bath, covered with a cover plate and cultured for 20 h (Supplementary Fig. S3A). Both the hot bath and the cover plate were maintained at 37 °C, which is the standard temperature for culturing *E. coli*.

Images of the culture chambers were obtained using a BZ-X800 microscope (Keyence, Osaka, Japan) with filters, GFP, and Cy5.

### Image analysis

The images were analyzed using OpenCV in the Python software. Because the regions near the PDMS walls of the two sides and the regions of the inlet and outlet may have unexpected effects, only the regions around the center of the incubation chamber were used for the analysis (Supplementary Fig. S3B). Fluorescent pixels were counted as bacterial regions.

### Simulation settings

The simulation was executed in a 256 × 256 square area. The simulations were performed using C and Python. The program flow is shown in Supplementary Fig. S4. Each bacterium has constant parameters “growth rate”, “death probability” and “energy consumption rate”, and a variable *E*_D_. “Growth rate” was fixed to 0.1 for all simulations. “Death probability” for each bacterium was set to 0.01. The initial cell number was set to 100 for all simulations. Boundary conditions are illustrated in Supplementary Figs. S5 B and C.

### Statistical analysis

Statistical analyses were performed using Python (SciPy and statsmodels libraries). Data are presented as mean ± standard error. Differences in bacterial growth under different conditions were evaluated using the Wilcoxon rank-sum test. A p-value < 0.05 was considered statistically significant. The sample size (N) is stated in the caption of each figure.

## Supplementary Information

Below is the link to the electronic supplementary material.

Supplementary Material 1

Supplementary Material 2

## Data Availability

The data analyzed during the current study available from the corresponding author on reasonable request.
