# Supplementary material for: Investigating the effects of soil microstructures on bacterial growth via microfluidic channels and an agent-based model
Source: Sci Rep. 2025 Nov 17;15:40257. doi: 10.1038/s41598-025-23995-9 (PMC12624013; doi:10.1038/s41598-025-23995-9)
Supplement: Supplementary file 1 — Supplementary Material 1 [file 41598_2025_23995_MOESM1_ESM.pdf]

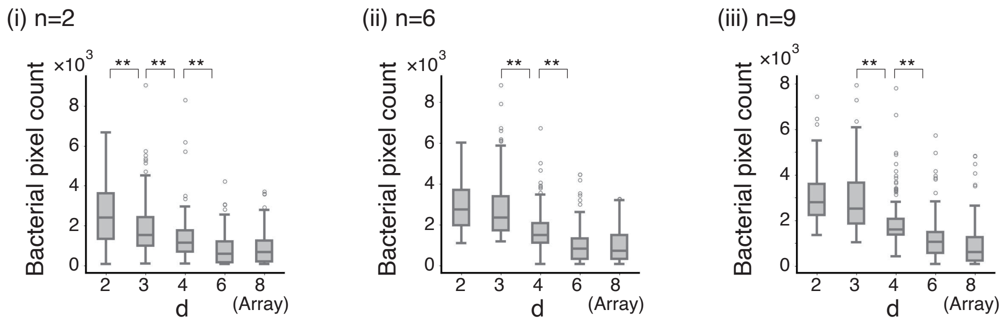

Figure S1. Boxplot of bacterial pixel counts for each arrangement with varying d at t = 150 for n=2, 6, 9. Results are based on 100 simulation runs. Statistical significance of the Wilcoxon rank-sum test is indicated by asterisks: **P < 0.01.

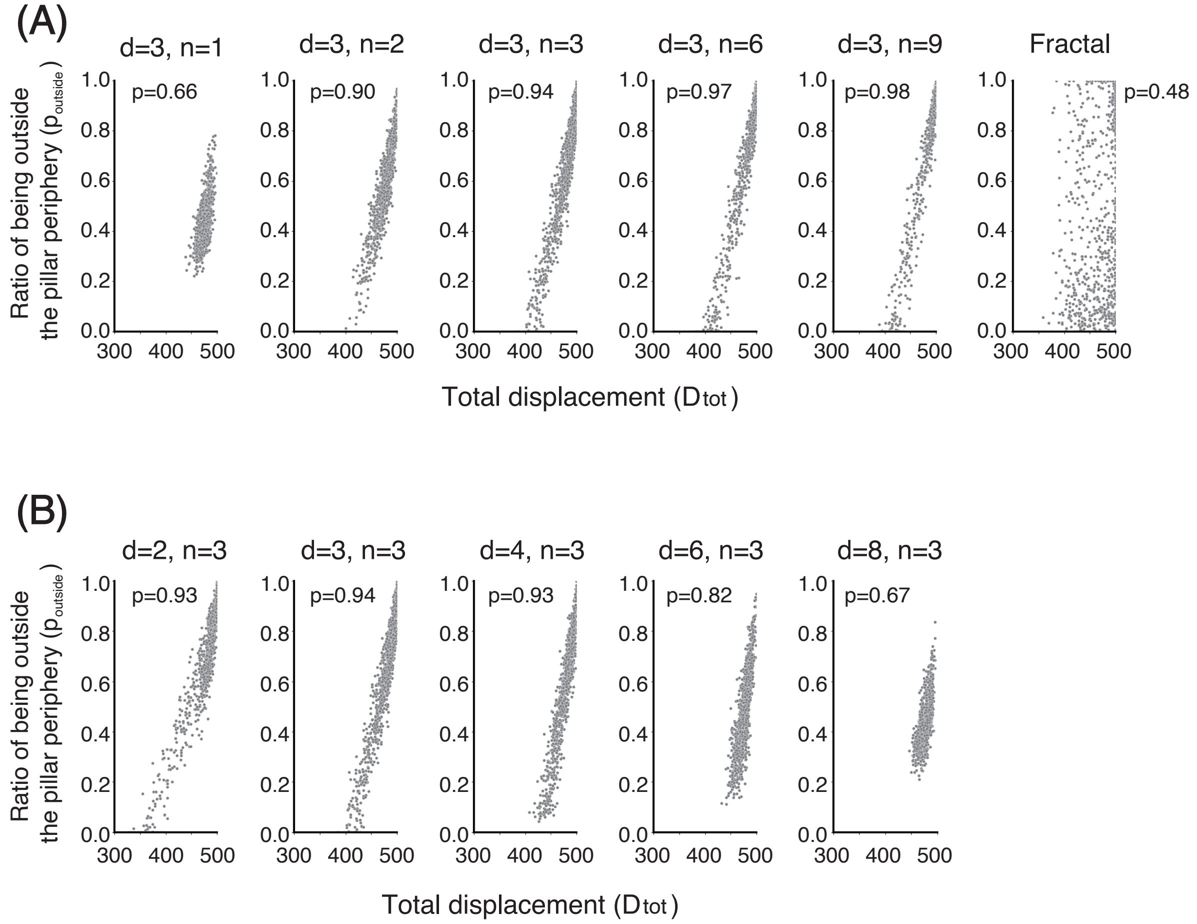

Figure S2. The correlation between $p_{outside}$ and D_{tot} for each arrangement.

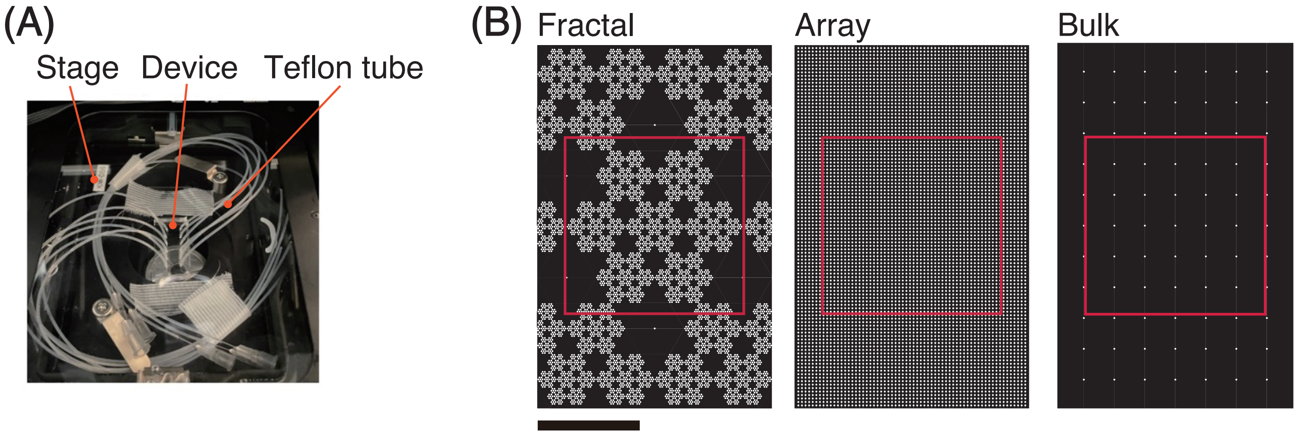

Figure S3. Experimental settings. (A) Photograph of the microfluidic device on the microscope stage in a hot bath. The device has six culture chambers (three fractal chambers and three array/six bulk chambers). TE tubes filled with the LB medium were connected to the inlets and outlets of the device. (B) The region enclosed by a square was used for analysis to prevent the unexpected influence of walls or inlets/outlets. Scale bar: 50 µm.

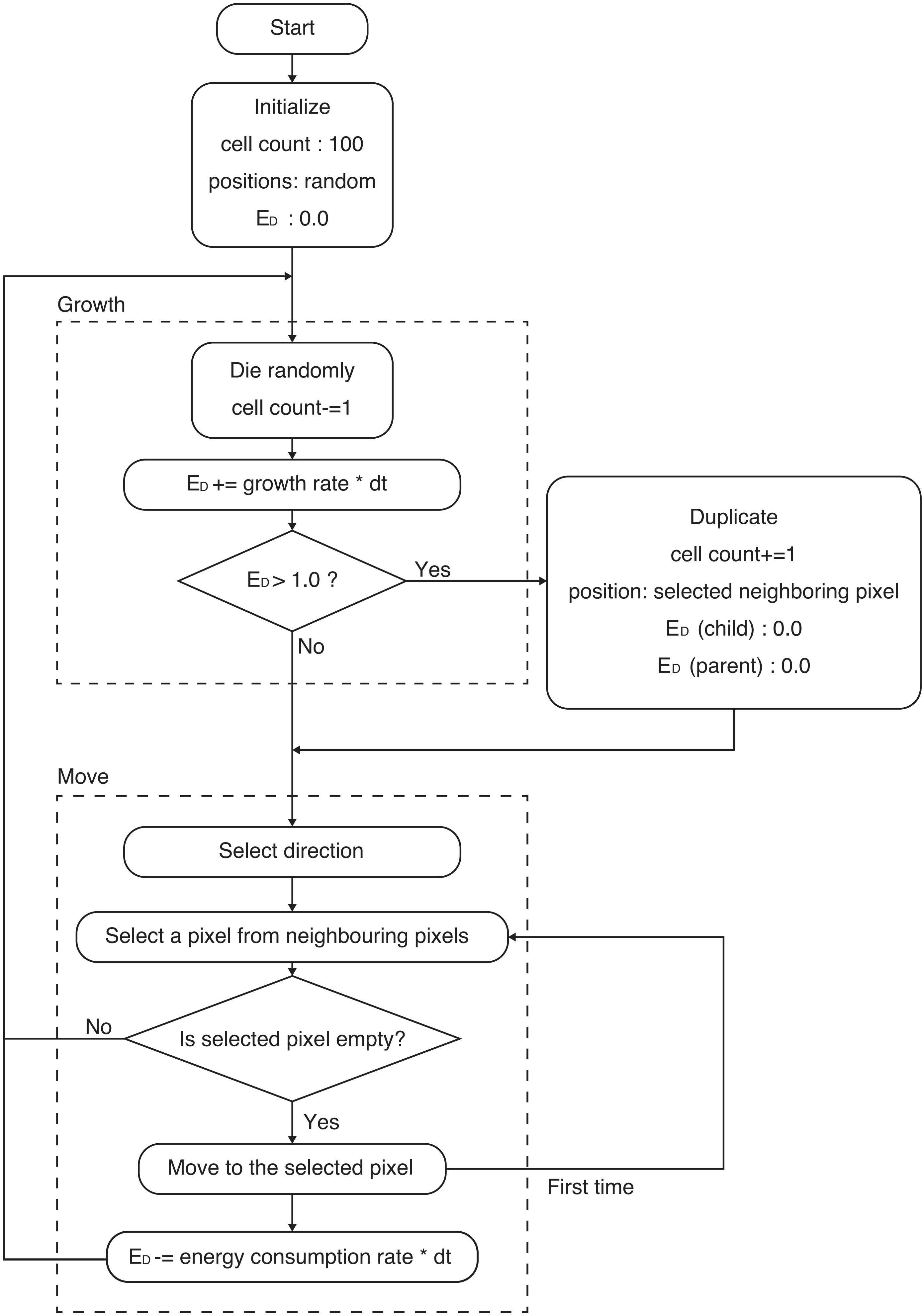

Figure S4. The illustration of program flowchart.

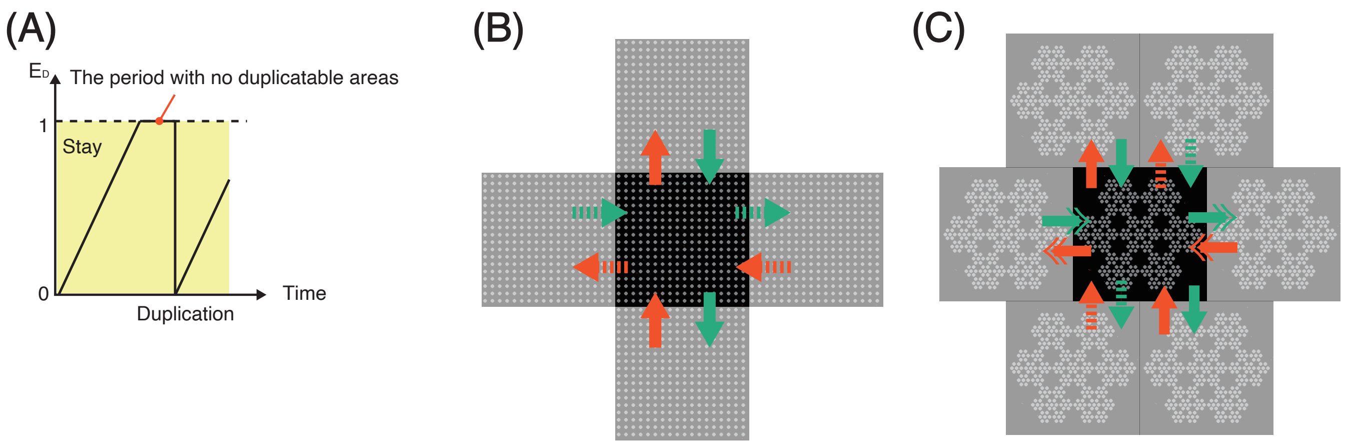

Figure S5. Simulation settings. (A) If there are no empty pixels around a bacterial pixel, the bacterial pixel cannot be duplicated, even if $E_D > 1$. (B) Boundary conditions of array and bulk arrangements. A bacterial pixel that moves and exceeds the left (right) boundary, will jump to the right (left) boundary. Similarly, a bacterial pixel that moves and exceeds the top (bottom) boundary jumps to the bottom (top) boundary. (C) Boundary conditions of the fractal arrangement. To represent the fractal condition, we modulated the boundary conditions of the array and bulk arrangements. A bacterial pixel that moves and exceeds the top (bottom) boundary jumps to the bottom (top) boundary and moves to the right by half the width of the simulation area. The horizontal boundary condition is the same as that of array and bulk arrangements. A bacterial pixel that moves and exceeds the left (right) boundary jumps to the right (left) boundary.
